# Evaluation of utilization of amplified blastocoel fluid DNA gel electrophoresis band intensity as an additional minimally invasive approach in embryo selection: A cross-sectional study

**DOI:** 10.18502/ijrm.v22i11.17823

**Published:** 2025-01-10

**Authors:** Fattaneh Khajehoseini, Zahra Noormohammadi, Poopak Eftekhari-Yazdi, Hamid Gourabi, Reza Pazhoomand, Shirzad Hosseinishenatal, Masood Bazrgar

**Affiliations:** ^1^Department of Biology, Science and Research Branch, Islamic Azad University, Tehran, Iran.; ^2^Department of Embryology, Reproductive Biomedicine Research Center, Royan Institute for Reproductive Biomedicine, ACECR, Tehran, Iran.; ^3^Department of Genetics, Reproductive Biomedicine Research Center, Royan Institute for Reproductive Biomedicine, ACECR, Tehran, Iran.; ^4^Medical Genetics Laboratory, Shiraz Fertility Center, Shiraz, Iran.; ^5^Department of Embryology, Shiraz Fertility Center, Shiraz, Iran.

**Keywords:** Aneuploidy, Blastocyst, Blastocoel fluid, Human embryo, Preimplantation genetic testing.

## Abstract

**Background:**

Embryo selection for transfer is critical in assisted reproduction. The presence of DNA in the blastocoel cavity of human blastocysts is assumed to be a consequence of common preimplantation chromosomal abnormalities.

**Objective:**

This study examined the relationship between the amount of blastocoel fluid (BF) DNA and the band intensity of amplified BF-DNA in gel electrophoresis, considering the influence of ploidy status.

**Materials and Methods:**

This cross-sectional study categorizes blastocysts into 2 groups based on the array comparative genomic hybridization results by trophectoderm biopsy -the euploid and aneuploid group. After morphological scoring, a biopsy of BF was performed for whole genome amplification, followed by an assessment of band intensity and BF-DNA quantification. The relationship between BF-DNA levels, band intensity, and ploidy status were analyzed.

**Results:**

The level of BF-DNA was higher in the aneuploid group compared to the euploid group, but the difference was not statistically significant (p = 0.2). We observed that the band intensity was affected by the ploidy status of the embryos, although this difference was not statistically significant (p = 0.059). Notably, electrophoresis band of all embryos with chromosomal loss was strong. No correlation was observed between embryo morphology and chromosomal ploidy (p = 0.8).

**Conclusion:**

Our findings indicate that BF-DNA band intensity on agarose gel is not currently applicable for preimplantation embryo selection. It seems that embryos with chromosomal loss are more prone to DNA release to BF. The disrelation between embryo morphology and ploidy status represents the necessity of minimally invasive embryo screening methods based on ploidy status.

## 1. Introduction 

To increase success in selecting a viable embryo for transfer, preimplantation genetic testing for aneuploidy (PGT-A) evaluates the ploidy status of embryos (1). During the formation of human blastocysts, simultaneously with the differentiation of embryonic cells into the inner cell mass (ICM) and the trophectoderm (TE), the Na ${\;}^{+}$ /K ${\;}^{+}$ pumps generate an osmotic gradient leading to the accumulation of water throughout the TE epithelium. The accumulation of fluid in the blastocoel cavity, where intercellular transport components, proteins, and DNA are found, leads to the expansion of the blastocyst (2). Following the discovery of suitable DNA for amplification and chromosomal condition determination from blastocoel fluid (BF) by Palini and colleagues, researchers' attention has been directed toward less invasive genetic evaluation of embryos, focusing on sampling from BF, sometimes referred to as “blastocentesis" (3). Efforts to noninvasively assess preimplantation embryos using BF-DNA have encountered significant challenges in isolating and amplifying retrieved DNA. These challenges may be the root of various factors such as low quantity and poor quality of BF-DNA, and technical difficulties in retrieving BF, particularly during the tubing stage where differences in the volume obtained can significantly impact the concentration of BF-DNA and may affect subsequent stages (4). The low quality and quantity of BF-DNA may be due to its likely degraded nature, possibly resulting from mechanisms of apoptosis or necrosis (5).

Studies have reported that human preimplantation embryos cultured in vitro, often display chromosomal mosaicism with predominant patterns of aneuploidy-diploidy resulting from errors in cell division during early development (6, 7). There is evidence suggesting that embryos may eliminate aneuploid cells through apoptosis as part of their self-correction mechanism (5, 8). If we accept that the BF-DNA is derived from apoptotic cells, then its use in PGT-A may be limited. To increase the copy number of BF-DNA for subsequent analysis, whole genome amplification (WGA) is required. Results of WGA have been reported with varying success rates (34.8–87.5%) in different studies (3, 5, 9–13). The reason for this variation could be attributed to factors such as the freshness or freezing of the embryos, the timing of sample collection, the chromosomal conditions of the samples, and the protocols of WGA used. Following successful WGA, various studies have reported different rates of concordance in chromosome analysis using samples of TE and BF-DNA (38.1–82.1%), these samples are generally derived from aneuploid embryos (4). Based on the latest findings, BF samples compared to TE or blastomeres show a significant rate of failed amplification (14, 15). A recent study comparing euploid and aneuploid blastocysts reported a significantly higher rate of unsuccessful amplification of BF-DNA in euploid blastocysts compared to aneuploid blastocysts. Their results suggested that the presence of DNA may be coordinated with the efforts of aneuploid embryos to survive (16).

There is very limited information about the band intensity after WGA and its relationship with the amount of DNA present in BF, which may be affected by the ploidy status of the embryo. This study aimed to evaluate the relationship between the quantity of BF-DNA, band intensity, and ploidy status.

## 2. Materials and Methods

### Ovarian stimulation

Ovarian stimulation was initiated with a daily dosage of 150 IU of Gonal-F (Merck Serono, Bari, Italy), a recombinant follicle-stimulating hormone, starting from the second day. Cetrotide (Merck, Idron, France), a gonadotropin-releasing hormone antagonist, at a dose of 0.25 mg/day was introduced on the 6 ${\;}^{\text{th}}$ day. Ovulation was induced by administering 0.1 mg of Decapeptyl (Ferring GmbH, Kiel, Germany), a gonadotropin-releasing hormone agonist, along with 250 μg of Ovitrelle (Merck Serono, Modugno, Italy), a recombinant human chorionic gonadotropin. Before performing intracytoplasmic sperm injection (ICSI), the cumulus cells enveloping the oocyte were isolated. Upon confirmation of fertilization through the observation of 2 pronuclei and a second polar body, the embryos were cultured until day 5, allowing them to develop into the blastocyst stage.

### Study design

This cross-sectional study included 40 frozen blastocysts donated by 20 couples who underwent PGT-A, through the analysis of 24 chromosomes using array comparative genomic hybridization (a-CGH) in TE cells. The study was conducted between September 2022 and December 2023 which included embryos that were not suitable for transfer. The indications for PGT-A were infertility and recurrent miscarriage. All embryos were anonymized at the time of donation with the participants' written informed consent. Based on the information obtained from embryos files for the a-CGH, the blastocysts were categorized into 2 groups euploid (n = 8), and aneuploid (n = 32). All embryos used in this study were previously frozen using the vitrification technique, which involved removing BF during the blastocyst stage. After thawing, only those blastocysts having the most degree of expansion were selected for blastocentesis. The embryos were excluded from the study due to their failure to undergo blastocyst expansion after thawing and their inability to form a blastocoel cavity. To address the objectives of this study, BFs from 40 blastocysts underwent WGA and after amplification, the quantification of BF-DNA was performed. The amplified samples were then subjected to electrophoresis on a 1.5% agarose gel. The Cochran formula was used to calculate the sample size, and statistical significance was set at p < 0.05. The blastocysts were evaluated using 4 variables (2 qualitative and 2 quantitative). The qualitative variables included ploidy status and band intensity on gel electrophoresis, while the quantitative variable was the embryo morphological score and the level of DNA content in the BF measured by a Qubit 2.0 Fluorometer (Thermo Fisher Scientific, USA). In the statistical analysis, the qualitative variables are converted into numerical variables. In order to quantitatively evaluate the results based on band intensity, inter- and intra-observation evaluation (kappa test) was done.

### Biopsy procedure

The frozen embryos were first warmed using a laboratory slow-thawing protocol. The embryo thawing was performed as previously described (17). In summary, thawing of the blastocysts was carried out by placing the cryotop in a sucrose-based thawing solution sequentially with concentrations of 1, 0.5, and 0.25 molar, each for 1, 3, and 3 min, respectively, at room temperature. The warmed blastocysts were placed into the washing solution (Ham's F-10 + 20% serum) for 4–5 rounds, they were then placed in the incubator (37 C, 5% O_2_, 5% CO_2_, and 90% N_2_) until they achieved expansion. The embryos were evaluated on day 5 using the Gardner and Schoolcraft grading system for morphological assessment. The embryos' morphological status was assessed using a scoring method that emphasizes the importance of blastocyst expansion, ICM, and TE to determine the most viable embryo, as detailed in the study by Rule et al. (18). The scoring formula used was: “weighted embryo morphology score = (expansion grade × 3) + (ICM grade × 2) + (TE grade × 1)”. BF biopsy was performed as previously described (2). In summary, BF samples aspirated from expanded blastocysts using an ICSI pipette, which minimizes the risk of potential damage and contamination with other cells embryos. A single BF aspiration was performed and precautions were taken to prevent the separation of any cellular material. The obtained fluids ( ∼ 0.001 μL) were transferred to 0.2 mL polymerase chain reaction tubes containing 2 µL of phosphate-buffered saline, which were kept in the cold shelf. The tip of the ICSI micropipette was broken into the tube to prevent material loss and immediately spun down after each biopsy. This method was repeated for all blastocysts, and the BF were immediately used for WGA. In the process of collecting BF adhered to established laboratory protocols. Regular air particle and microbiological assessments are conducted within the in vitro fertilization (IVF) laboratory unit, particularly in the areas and hoods designated for embryo culture, to ensure compliance with international sterile condition standards. Operators utilized gloves, masks, laboratory coats, and hairnets during sample retrieval and storage. Sterile hoods are used to prepare each culture dish, with the culture drops covered in mineral oil. Additionally, all equipment, including dishes, tips, and pipettes, was replaced before aspirating BF from each sample.

### WGA and BF-DNA quantification

DNA amplification using REPLI-g WGA kit (QIAGEN, USA) was performed in a class II laminar flow hood, following the manufacturer's instructions. In summary, 2.5 µL of each sample was mixed with kit components to reach a total volume of 50 µL and then incubated at 30 for 8 hr, subsequently, the samples were heated to 65 for 3 min to deactivate the DNA polymerase. The bands intensity of the electrophoresis after amplification was examined by loading 5 µL of the final WGA product onto a 1.5% agarose gel and running it for 50 min. A single human blastomere WGA with strong band intensity was considered as positive control. Strong amplification bands with similar intensity to the positive control were observed, while failed and weak amplification was considered similar to the negative control on the gel. The samples were loaded in 3 replicates. In order to minimize technical errors, the results of gel electrophoresis were interpreted by 2 investigators without information about ploidy status. DNA concentration was measured using the Qubit 2.0 Fluorometer (Thermo Fisher Scientific, USA) and the Qubit dsDNA HS assay kit (Thermo Fisher Scientific, USA), following the manufacturer's instructions. This kit enables quantification through the exclusive binding of a fluorescent dye to dsDNA, even in the presence of other biological molecules. It determines the amount of dsDNA by measuring fluorescence intensity by plotting a standard curve. The quantity of BF-DNA in each sample was measured using 1 µL of the final WGA product.

### Ethical Considerations

This study was performed in line with the principles of the Declaration of Helsinki. Approval was granted by the Royan Institutional Research Ethics Committee, Tehran, Iran (Code: IR.ACECR.ROYAN.REC.1400.094). Written informed consent was obtained from all participants involved in the study.

### Statistical Analysis

Statistical analysis was performed using GraphPad Prism 9.9.0 software (GraphPad Software, San Diego, CA, USA) and IBM SPSS Statistics (Version 24). The normality of the variables within the groups was checked by the Kolmogorov-Smirnov test. In the presentation of data, qualitative variables were shown as numbers and percentages, while quantitative variables were detailed through their mean ± standard deviation, and interquartile range. The Mann-Whitney test was used to compare the 2 groups. The Chi-squared test and Fisher's exact test were used to compare qualitative variables. Kappa coefficient accuracy assessment was calculated to evaluate the agreement between 2 qualitative variables. Differences were considered significant if p < 0.05.

## 3. Results

**Figure 1:**
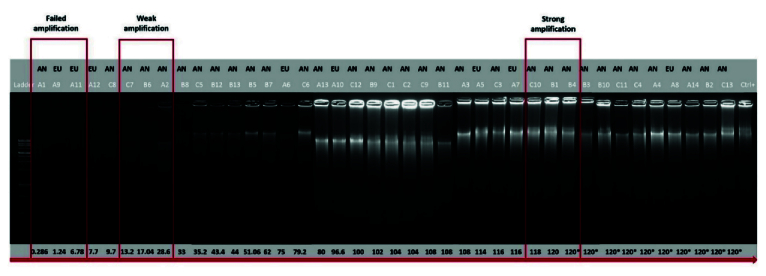
Quality control blastocoel fluid (BF) DNA amplification from 40 BF by 1.5% agarose gel electrophoresis. BFs were run on agarose gel based on the increase in DNA concentration read by Qubit. Successful amplification, failed and weak amplification showed strong band, no band, and weak band on the gel, respectively. *BF-DNA concentrations > 120 ng/µl. EU: Euploidy, AN: Aneuploidy.

A total of 40 BF were collected from blastocysts after expansion and sending for WGA (Table I). These BF originate from blastocysts that were previously subjected to TE biopsy for a-CGH analysis of each of the 24 chromosomes (maternal age 37.51 ± 6.19 yr). Based on the a-CGH results, the embryos have been classified into euploid (n = 8), and aneuploid (n = 32) groups. The band intensity of BF-DNA amplifications on the electrophoresis gel was categorized into failed/weak amplification (weak) and successful amplification (strong), (Figure 1). Overall, 9 embryos were classified in the weak band group (9/40, 22.5%) and 31 in strong band group (31/40, 77.5%) (Table I). Partially, 92.5% of the BF samples (37/40) showed bands, while 7.5% of the BF samples (3/40) that belonged to weak band group, showed no bands on the gel agarose (Figure 1). In the euploid group, 50% of the samples (4/8) displayed weak bands, while the remaining 50% (4/8) showed strong bands. When comparing the 2 groups of euploid and aneuploid blastocysts, successful amplification (strong band) was less frequent in euploid blastocysts (4/8, 50%) compared to the aneuploid group (27/32, 84.37%). In contrast, unsuccessful amplification (weak band) of BF-DNA was more prevalent in euploid blastocysts (4/8, 50%) than in aneuploid blastocysts (5/32, 15.63%). Table II presents the percentages reported according to the grouping of euploid and aneuploid embryos. Further analysis focused on the aneuploid blastocysts, categorizing them based on chromosomal gains and losses. In the aneuploid group, 19 blastocysts showed chromosomal gain (Table I, embryos 9–27), while 13 showed chromosomal loss (Table I, embryos 28–40). Among the blastocysts with chromosomal gains, 26.32% (5/19) demonstrated weak bands, whereas 73.68% (14/19) exhibited strong bands. Notably, all aneuploid blastocysts with chromosomal losses presented strong bands on agarose gel (13/13, 100%), (Table III).

The results indicated that the relationship between band intensity on gel electrophoresis (weak and strong) in the euploid and aneuploid groups was not statistically significant (p = 0.059), (Table III). The concentration of BF-DNA after WGA was investigated in euploid and aneuploid groups for each of the 40 samples.

**Figure 2:**
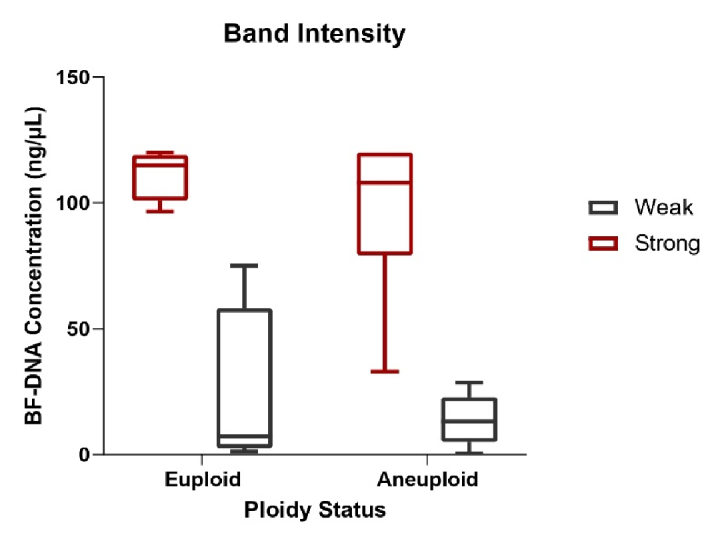
Box plot illustrating the distribution of the number of samples with weak and strong band intensities, based on BF-DNA concentration, categorized into euploid and aneuploid groups. BF: Blastocoel fluid.

The measurable concentration of BF-DNA was obtained in 30 samples. Values > 600 ng/ml, which are read as “sample too high" by Qubit, were considered equal to 120 (the largest value read by the Kit) in our study. BF-DNA concentrations > 120 ng/µl (nonmeasurable) were obtained for 10 blastocysts (10/40, 25%), which are recognizable with an asterisk in table I. The percentage of BF samples that tested positive for BF-DNA (40/40, 100%) was recorded along with an average concentration of 75.22 ng/µL between the 2 groups. Comparison between the 2 blastocyst groups showed that the average amount of BF-DNA in aneuploid blastocysts was higher than in euploids (83.27 ng/µL vs. 67.16 ng/µL); however, this difference was not statistically significant (p = 0.2, Table II). The distribution of gel electrophoresis band intensity data, categorized by euploid and aneuploid groups based on BF-DNA concentration, was notable. A greater number of samples with weak bands were observed in the euploid group, while more samples with strong bands were found in the aneuploid group, based on BF-DNA concentration. However, this difference was not statistically significant (p > 0.05, Figure 2).

The Kappa coefficient for assessing agreement between 2 observers regarding band intensity (weak and strong) was 1.000, indicating perfect agreement (p < 0.001). The Kappa coefficient for band intensity between euploid and aneuploid groups was -0.067, reflecting a lack of significant agreement (p = 0.618).

Embryo morphology scores ranged from 9 to 27, with a mean of 18.75 in the euploid group compared to 18.78 in the aneuploid (Table I, II). The morphology score did not show a statistically significant relationship with the aneuploidy (p > 0.05). No significant correlation was observed between embryo morphology score ploidy status and band intensity in euploid and aneuploid groups (p = 0.8), (Table II).

**Table 1:** Details of the included embryos

**Embryo**	**Karyotype**	**Category**	**Band intensity**	**Blastocoel fluid-DNA (ng/µL)**	**Morphology score**
**1**	46, XX	Euploid	Weak	1.24	15
**2**	46, XX	Euploid	Weak	6.78	24
**3**	46, XX	Euploid	Weak	7.7	18
**4**	46, XX	Euploid	Weak	75	18
**5**	46, XY	Euploid	Strong	96.6	24
**6**	46, XX	Euploid	Strong	114	15
**7**	46, XX	Euploid	Strong	116	18
**8**	46, XX	Euploid	Strong	120*	18
**9**	Gain, 47, XXY	Aneuploid	Weak	0.28	15
**10**	Gain, 47, XY, +12	Aneuploid	Weak	17.04	21
**11**	Gain, 47, XXY	Aneuploid	Weak	28.6	18
**12**	Gain, 47, XY, +15, 20	Aneuploid	Strong	35.2	17
**13**	Gain, 47, XY, +16	Aneuploid	Strong	51.06	18
**14**	Gain, 47, XXY	Aneuploid	Strong	80	18
**15**	Gain, 47, XY, +13	Aneuploid	Strong	108	16
**16**	Gain, 47, XXY	Aneuploid	Strong	120*	24
**17**	Gain, 47, XXY	Aneuploid	Strong	120*	20
**18**	Gain, 48, XY, +15, 20	Aneuploid	Strong	102	21
**19**	Gain, 48, XX, +17, +19	Aneuploid	Strong	120*	24
**20**	Gain, 49, XX, +11, 16, 20	Aneuploid	Weak	9.7	18
**21**	Gain, 49, XX, -7, 11, 15, 18	Aneuploid	Strong	104	18
**22**	Gain, 49, XX, +16, 17, 18, 21, 13	Aneuploid	Strong	118	15
**23**	Gain, 51, XXY, +10, 14, 15, 20	Aneuploid	Strong	100	18
**24**	Gain, 51, XXY, +(5, 9)x2	Aneuploid	Strong	120*	24
**25**	Gain, 54, XXXY, + 9, 10, 11, 12, 15, 21	Aneuploid	Weak	13.2	15
**26**	Gain, 55, XXY, +(2, 17, 19, 20)x2	Aneuploid	Strong	116	22
**27**	Gain, 58, XY, +4, 7, 13, 16, 19, 20x2	Aneuploid	Strong	120*	21
**28**	Loss, 45, XX, -20	Aneuploid	Strong	33	27
**29**	Loss, 45, XY, -16	Aneuploid	Strong	43.4	24
**30**	Loss, 45, XX, -15	Aneuploid	Strong	44	24
**31**	Loss, 45, XY, -22	Aneuploid	Strong	62	11
**32**	Loss, 45, XY, -16	Aneuploid	Strong	108	17
**33**	Loss, 45, XY, -1	Aneuploid	Strong	108	19
**34**	Loss, 45, XX, -13	Aneuploid	Strong	120	18
**35**	Loss, 45, XX, -16	Aneuploid	Strong	120*	21
**36**	Loss, 45, XX, -16	Aneuploid	Strong	120*	18
**37**	Loss, 45, XX, -13	Aneuploid	Strong	120*	17
**38**	Loss, 41, XX, -5, 7, 11, 12, 13	Aneuploid	Strong	120*	9
**39**	Loss, 41, XY, -5, 8, 10, 11, 15	Aneuploid	Strong	79.2	18
**40**	Loss, 41, XO, -6, 19, 20, 21, Y	Aneuploid	Strong	104	15
The karyotype column presents array comparative genomic hybridization results from trophectoderm biopsy, the amount of blastocoel fluid DNA read by Qubit, and describes the band intensity in agarose gel electrophoresis (weak and strong) in human blastocysts in 2 chromosomal categories, euploid and aneuploid. Samples were sorted based on ploidy status. *BF-DNA concentrations > 120 ng/µL					

**Table 2:** Comparison of band intensity, karyotype, BF-DNA concentration, and morphology score between euploid and aneuploid groups

**Variables**		**Ploidy status**			
		**Euploid (n = 8)**	**Aneuploid (n = 32)**	**Pearson Chi-square |**
**Mann-Whitney**	**P-value**			
Band intensity*					
	**Weak**	4 (50)	5 (15.6)		
	**Strong**	4 (50)	27 (84.4)	4.337^a^	0.059 ${\;}^{\text{c}}$
Karyotype*					
	**Gain**	-	19 (59.4)		
	**Loss**	-	13 (40.6)	**-**	**-**
BF-DNA concentration**		67.16 ± 18.81 85.5 (7.01–115.5)	83.27 ± 7.320 104 (43.55–120)			94.5^b^	0.252 ${\;}^{\text{d}}$
Morphology score**		18.75 ± 1.235 18 (15.75–22.5)	18.78 ± 0.689 18 (17–21)	123.0^b^	0.863 ${\;}^{\text{d}}$
*Data presented as n (%). **Data presented as Mean ± SD, median (interquartile range). a: Pearson Chi-square, b: Mann-Whitney, c: Chi-square test, d: Mann-Whitney test, BF: Blastocoel fluid					

**Table 3:** Comparison of band intensity in aneuploid group with gain and loss of karyotype

	**Aneuploid group**			
<brow>-2</erow> **Band intensity**	**Gain (n= 19)**	**Loss (n = 13)**	**Pearson Chi-square**	P-value*
**Weak**	5 (26.3)	0 (0)				
**Strong**	14 (73.6)	13 (100)	4.055	0.064
Data presented as n (%). *Chi-square test				

## 4. Discussion

To develop less invasive methods in assisted reproduction, we focused on quantifying the amount of DNA retrieved from the BF of expanded euploid and aneuploid blastocysts, previously identified through TE chromosome analysis. We evaluated the relationship between amplified BF-DNA quantity and the bands intensity of gel electrophoresis affected by the ploidy status of blastocysts.

In summary, the ratio of samples containing DNA in BF after WGA, as read by Qubit, was 100% (40/40), while the ratio of samples exhibiting bands on the gel was 92.5% (37/40). Notably those 3 samples with the lowest amounts of BF-DNA, as measured by Qubit, showed no bands on the gel; 2 of these samples were euploid (Figure 1). The differences in band intensity on the electrophoresis gel between the euploid and aneuploid blastocyst groups suggest that ploidy status affects band intensity, with a noticeable increase in the number of strong bands in the aneuploid group compared to euploid group (27/32, 84.37% vs. 4/8, 50%). However, this difference did not reach statistical significance (p = 0.059), likely due to the limited sample size in our study (Table II).

What becomes increasingly evident is that the presence of BF-DNA in the blastocoel cavity could be a result of the embryo's efforts to correct aneuploidy through mechanisms such as apoptosis or necrosis of aneuploid cells, especially in mosaic embryos, providing a chance for their viability and development continuation (5, 8, 18, 19). However chromosomal errors, especially in complex aneuploidy conditions, are unlikely to be rectified effectively in embryonic response to aneuploidy (20). Tobler and colleagues reported that by assessing the concordance of ploidy status among blastomere, TE, and BF samples, embryos with aneuploidy at the cleavage stage likely differentiate into euploid blastocysts using a mechanism where aneuploid nuclei are pushed to the blastocoel cavity (5). The results of a study in the preimplantation chimeric mouse model demonstrated the selective elimination of aneuploid cells via apoptosis mechanism (8). Next-generation sequencing analysis of BF revealed that the predominant population of DNA fragments in the blastocoel cavity ranged in size from 160–220 bp, similar to what is found in circulating blood plasma (11).

Currently, if these findings accurately reflect biological reality, we expect the amount of DNA released into the blastocoel cavity to be influenced by the chromosomal conditions of the embryos which may, in turn, affect the band intensity. Notably, all aneuploid blastocysts with chromosomal loss, regardless of the involved chromosome or the number of losses, displayed strong bands on the gel (Table I, III). This finding may be associated with an increased likelihood of apoptosis and release of DNA into the blastocoel cavity in embryos with chromosomal loss. As mentioned, an increase in BF-DNA was observed in the aneuploid group compared to the euploid group; however, it was not statistically significant (p = 02).

While there is limited information regarding the impact of ploidy status on BF-DNA levels and successful amplification, Magli and colleagues indicated a significantly higher incidence of unsuccessful amplification in euploid blastocysts compared to aneuploid ones. Their study found that DNA presence in BF correlates with blastocyst ploidy status and implantation potential, suggesting that prioritizing TE-euploid blastocysts with BF-failed amplification could enhance embryo selection in both fresh and vitrified IVF cycles (16). Based on these findings, we analyzed the amount of BF-DNA in the blastocoel cavity after WGA and its relationship with the band intensity on electrophoresis gel, which we assume reflects chromosomal conditions. However, in our study, as the embryos were only available for research purposes, we were unable to track clinical outcomes and, therefore, could not assess the relation between band intensity and implantation and live birth outcomes. Our focus was primarily on investigating the relationship between ploidy status and BF-DNA band intensity, aiming to explore whether band intensity following BF-DNA amplification is applicable for embryo screening and selection with less chance of aneuploidy as a prevalent status in preimplantation stage.

Our analysis revealed a higher incidence of failed and weak amplifications in euploid blastocysts compared to aneuploid blastocysts (50% vs. 15.62%); however, this difference was not statistically significant. In other words, embryos with aneuploidy have higher levels of DNA in their blastocoel cavity and exhibit stronger band intensity on electrophoresis gels. This suggests that exposure to aneuploidies results in increased shedding of BF-DNA, likely due to a higher incidence of apoptosis in cells affected by chromosomal abnormalities. This aligns with our study's results, which demonstrated a higher average amount of DNA in BF in the aneuploid group compared to the euploid group (83 ng/µL vs. 67 ng/µL); however, this difference did not show statistical significance (p = 02). This may be due to several factors: the small sample size in our study groups, particularly the limited access to a larger number of euploid embryos, and the Qubit reads, which classified concentrations > 120 ng/µL as equal to 120 ng/µL. Importantly, 90% (9 out of 10) of the samples exceeding 120 ng/µL were found in the aneuploid group, suggesting that the actual BF-DNA levels in this group may be higher than the reported amounts. Therefore, utilizing a bioanalyzer instead of Qubit could improve the accuracy of BF-DNA quantification. A very low volume of BF (in nanoliter range) can be challenging for testing purposes. Although a professional expert embryologist performed our sample collection, the tip of the ICSI micropipette was broken into the BF tube to prevent material loss.

The other limitations of our study, which may have influenced the findings, are as follows: our assessment was based on BF-DNA analysis while the embryos' karyotypes were obtained from TE biopsy. Although TE biopsy, combined with advanced methods like a-CGH, can provide valuable insights, the presence of mosaicism means these results may not accurately represent the genetic makeup of the whole embryo. Therefore, even if a TE biopsy shows a euploid result with a high likelihood of viability, it may ultimately not lead to successful implantation or live birth. Furthermore, since the embryos were only available for research purposes, we were unable to follow up on pregnancy or birth outcomes. The freezing of blastocysts and the potential for cell damage during the freezing process, could result in the release of cellular DNA into the blastocoel cavity. However, this likelihood is minimal as we utilized the vitrification method for sample preservation.

At the beginning of the freezing process, apoptotic pathways start cell death process with changes in gene expression, exposure to low temperatures during various freezing methods plays a crucial role in activating these apoptotic pathways in mammalian oocytes and embryos (21). Therefore, it seems that the selected method in embryo freezing (slow freezing or vitrification) is an effective factor in reducing or increasing apoptosis and necrosis of blastomeres; however, few studies have investigated the apoptosis of embryos after slow freezing and vitrification, especially in humans. A study showed the low possibility of damage to the embryo in the conditions of vitrification compared to slow freezing, and the similarity of the results between vitrified and fresh embryos and even higher fertility rates compared to the transfer of fresh embryos and the possibility of survival (22). Embryos vitrification for storage and the preservation of their developmental capacity is a critical aspect in assisted reproductive technologies and removal of BF is a common clinical practice in IVF as the most effective method of embryo cryopreservation and storage for subsequent stages without imposing additional costs on patients or clinics. Therefore, the use of BF can remain a research area.

Currently, embryo assessment based on morphological features is widely regarded as the gold standard among embryologists. However, this method alone lacks sufficient accuracy regarding unwanted transfer of aneuploid embryos that leads to assisted reproductive technologies failure. Studies involving large numbers of embryos indicate that aneuploidy is independent of morphology, meaning that embryos with good morphological characteristics may still exhibit single or multiple aneuploidies (23–25). Consequently, it is not surprising that our study found no correlation between embryo morphology scores and band intensity in both the euploid and aneuploid groups. Given this lack of relationship between morphology and ploidy status, there is an ongoing need to enhance minimally invasive embryo selection methods that can more accurately reflect the ploidy status of preimplantation embryos.

In the present study, no significant relationship was observed between BF-DNA quantity and embryo ploidy status, or between gel electrophoresis band intensity and ploidy status. One of the reasons is the small sample size of euploid cases, where no clear pattern was observed (50% strong bands vs. 50% weak bands). Based on these findings, our study indicates that this method is currently not reliable for embryo selection. Due to limited availability of human embryos for this study, future research using larger sample sizes is recommended.

## 5. Conclusion

In this study, we found that the band intensity, which is associated with the amount of DNA released in the blastocoel cavity, did not significantly correlate with the chromosomal status of preimplantation embryos. As all embryos with chromosomal loss represented strong bands on gel electrophoresis, such aneuploid embryos seem to be more prone to DNA release to BF. However, the use of band intensity for assessing preimplantation embryo quality is currently not applicable. The disrelation between embryo morphology and ploidy status represents the necessity of minimally invasive embryo screening methods based on ploidy status. Considering, the significance of noninvasive methods in identifying optimal embryos with the most implantation potential, we recommend larger studies with a greater number of both fresh and frozen euploid and aneuploid embryos along with follow-up on clinical outcomes for more consistent evaluation of potential utilization of amplified BF DNA gel electrophoresis band intensity as an additional minimally invasive approach in embryo selection.

##  Data Availability

Data supporting the findings of this study are available upon reasonable request from the corresponding author.

##  Author Contributions

F. Khajehoseini, M. Bazrgar, Z. Noormohammadi, H. Gourabi, and P. Eftekhari-Yazdi contributed to the conception and design of the work and interpretation of data. F. Khajehoseini, P. Eftekhari-Yazdi, Sh. Hosseinishenatal, and R. Pazhoomand performed the experiments. F. Khajehoseini, M. Bazrgar, Z. Noormohammadi, and R. Pazhoomand analyzed the data. F. Khajehoseini, M. Bazrgar, and H. Gourabi drafted and revised it critically for important intellectual content. M. Bazrgar provided the funds. All co-authors approved the final version to be published and agreed to be accountable for all aspects of the work.

##  Acknowledgments

The authors are grateful to all of the patients who agreed to donate their embryos for research. All the financial support for this project was provided by Royan Institute, Tehran, Iran (project code: 99000154). Also, we have not used artificial intelligence in any way (translation, revision, grammar check, etc.).

##  Conflict of Interest

The authors declare that there is no conflict of interest.
